# Increased platelet activating factor levels in chronic spontaneous urticaria predicts refractoriness to antihistamine treatment: an observational study

**DOI:** 10.1186/s13601-019-0275-6

**Published:** 2019-07-17

**Authors:** Bastsetseg Ulambayar, Eun-Mi Yang, Hyun-Young Cha, Yoo-Seob Shin, Hae-Sim Park, Young-Min Ye

**Affiliations:** Department of Allergy and Clinical Immunology, Ajou University School of Medicine, 164 Worldcup-ro, Yeongtong-gu, Suwon, 443-721 Korea

**Keywords:** Platelet activating factor, Platelet activating factor acetylhydrolase, Chronic spontaneous urticaria

## Abstract

**Background:**

Platelet activating factor (PAF) is an endogenous, active phospholipid released from inflammatory cells, platelets, and endothelial cells, and is involved in the regulation of immune responses. Degradation of PAF by PAF acetylhydrolase (PAF-AH) has been shown to be associated with anaphylaxis, asthma, and peanut allergy. The purpose of this study was to investigate relationships among clinical parameters, including urticaria severity and treatment responsiveness, and PAF and PAF-AH levels in sera from patients with chronic spontaneous urticaria (CSU).

**Methods:**

Serum PAF and PAF-AH levels were measured by enzyme-linked immunosorbent assay in 283 CSU patients and 111 age- and sex-matched, healthy normal controls (NCs). Urticaria severity was evaluated by urticaria activity score over 7 days (UAS7). Within 3 months after measuring PAF levels, patients whose urticaria was not controlled by antihistamine treatment were classified as histamine receptor 1 antagonist (H1RA) non-responders.

**Results:**

Serum PAF levels were significantly higher in CSU patients than in NCs (median 4368.9 vs. 3256.4 pg/ml, p = 0.015), while serum PAF-AH levels were significantly lower in CSU patients (105.6 vs. 125.7 ng/ml, p = 0.001). H1RA non-responders had higher levels of PAF in their sera than H1RA responders. A generalized linear model revealed that a higher UAS7 score (odds ratio 1.023, p = 0.024) and a PAF level ≥ 5000 pg/ml (1.409, p < 0.001) were significant predictors of a poor response to H1RA treatment.

**Conclusions:**

Compared with NCs, CSU patients, particularly those with H1RA refractoriness, showed significant increases in serum PAF levels and decreases in PAF-AH. Therapies modulating PAF and PAF-AH levels could be effective in patients with CSU refractory to antihistamines.

## Background

Platelet activating factor (PAF, 1-O-alkyl-2-acetyl-sn-glycero-3-phosphocholine) is a bioactive phospholipid that plays a critical role in immune and inflammatory conditions [1]. It has been found to be produced by most immune cells, including basophils, eosinophils, mast cells, lymphocytes, and macrophages, as well as platelets and endothelial cells [1]. PAF binds to its specific receptor PAFR, a G protein-coupled receptor expressed on several immune cells, including mast cells and basophils [2]. Metabolism of PAF is regulated by the enzyme PAF-acetylhydrolase (PAF-AH), which is responsible for PAF degradation. PAF has been found to be involved in the pathogenesis of cardiovascular diseases, psoriasis, sepsis, and allergic diseases, including asthma, allergic rhinitis, anaphylaxis, and urticaria [1, 3–5].

Chronic urticaria (CU) is a chronic inflammatory skin disorder characterized by recurrent itchy wheals lasting longer than 6 weeks. CU is classified into inducible urticaria (induced by specific physical triggers) and chronic spontaneous urticaria (CSU) [6]. Resulting in vasodilatation, plasma extravasation, and inflammatory cell migration to urticarial lesions, mast cells and basophils release various inflammatory mediators, including histamine, protease, cytokines, chemotactic factors, arachidonic acid metabolites, neuropeptides, and PAF [6]. Accordingly, recent guidelines recommend non-sedating H1 antihistamines (histamine1 receptor antagonist, H1RA) for first-line treatment of CSU [6]. Except for histamine, however, the roles of inflammatory mediators in the pathogenesis of CSU have yet to be thoroughly investigated.

Research suggests that PAF plays an important role in anaphylactic reactions, being correlated with the severity thereof [1, 7]. Interestingly, in severe anaphylaxis, PAF levels have been found to be inversely correlated with PAF-AH levels [8]. The present study aimed to investigate associations of PAF and PAF-AH levels in sera in relation to clinical implications in CSU patients.

## Methods

### Study subjects

In total, 283 patients with CSU who had urticaria symptoms for more than 6 weeks and 111 healthy normal controls (NCs) were recruited. Inclusion criteria were age ≥ 19 years and having urticaria symptoms almost daily, such as wheals and itching or angioedema, for at least 6 weeks. Patients with other chronic skin diseases and those with clinical evidence of urticarial vasculitis or inducible (physical stimuli or cholinergic or exercise) urticaria were excluded. Sera from healthy normal controls were supplied by the Ajou University Human Bio-Resource Bank. Normal controls (NCs) were confirmed by the Biobank as not having any previous history of inflammatory or allergic skin disease or urticaria.

UAS7 (urticarial activity score over 7 days), a weekly urticaria activity score, was utilized to assess disease activity [6]. Body mass index (BMI) was calculated as weight/height (kg/m^2^). Aspirin intolerance was diagnosed based on a history of recurrent urticaria after aspirin/non-steroidal anti-inflammatory drug (NSAID) ingestion or positive results of oral challenge to aspirin, as previously described [9]. Written informed consent was collected from all study subjects. The study was approved by the Institutional Review Board of Ajou University Medical Center (AJIRB-BMR-SMP-18-74).

### Assessment of clinical parameters

Autologous serum skin test (ASST) was performed and evaluated in accordance with reported guidelines [10]. Atopy was defined as at least one positive skin test result to common allergens or increased serum levels of specific Immunoglobulin E (IgE) to *Dermatophagoides farina*, *D.pteronyssinus*, or other common inhalant allergens. Anti-nuclear antibody (ANA) was detected using an indirect fluorescent antibody technique (Fluoro HEPANA test, Medical & Biological Laboratories, Nagoya, Japan). Total IgE and eosinophilic cationic protein (ECP) were measured by the ImmunoCAP system (Pharmacia Diagnostics, Uppsala, Sweden) according to the manufacturer’s instruction. Total cholestrol, triglyceride, and high and low density lipoproteins (HDL and LDL) were measured by enzymatic colorimetric assay using a TBA-200FR anlalyser (Toshiba, Tokyo, Japan). Complement 3 and 4 levels were measured using an immunoturbidimetric assay (Roche Hitachi Cobas C system, Rotkreuz, Switzerland). Antihistamine doses were described as equivalent doses of loratadine (mg/day).

### Serum PAF and PAF-AH levels

Before collecting serum samples, antihistamines were withdrawn for at least 5 days. Serum samples were stored at − 70 °C until ready for use. Serum PAF and PAF-AH levels were measured by commercial enzyme-linked immunoabsorbent assay (ELISA) kits (LifeSpan BioSciences, Inc., Seattle, WA, USA and R&D systems Inc., Minneapolis, MN, USA, respectively). The serum samples were diluted based on the detection ranges of PAF (78.3–5000 pg/ml) and PAF-AH (0.781–50 ng/ml).

### Statistical analysis

Statistical analyses were performed using IBM SPSS, version 25 for Windows (SPSS Inc., Chicago, IL, USA). Data are presented as a mean ± standard deviation for clinical parameters and as a median (interquartile range [IQR]) for PAF and PAF-AH. As distributions of total IgE levels in both CSU and NC groups, were highly right-skewed, total IgE were logarithmically transformed (base 10) for statistical analyses. Statistical significance was assessed by Mann–Whitney U-test or Fisher’s exact test, and correlation analyses were conducted by Spearman’s correlation. The level of PAF ≥ 5000 pg/mL was obtained as the cut-off for detecting non-responders to antihistamines based on receiver operator characteristics (ROC) curve analysis (area under the curve 0.642, p < 0.001). A generalized linear model was applied to determine the effects of PAF and PAF-AH on treatment response in CSU patients.

## Results

### Characteristics of study subjects

The clinical characteristics of study subjects are summarized in Table 1. We enrolled 283 CSU patients (42.4 ± 11.7 years old, 184 females) and 111 age- and sex-matched NCs (43.9 ± 11.3 years old, 73 females). BMI was not different between the CSU patients and NCs (23.5 ± 3.9 vs. 23.1 ± 2.8 kg/m^2^, p = 0.864). The mean urticaria duration was 26.1 months, and the mean UAS7 score was 24.4 ± 10.3. The prevalences of angioedema and aspirin intolerance were 45.7% and 25.5% in CSU patients, respectively. Among the CSU patients, 30.2% had a positive response to ASST, and 21.4% were positive on ANA. The atopy rate was 54.2%, and the mean level of log-transformed total IgE (kU/L) was 2.09 ± 0.5 in CSU patients. Mean serum ECP level was higher in CSU patients than in NCs (21.7 ± 18.9 vs. 12.7 ± 9.6 kU/L, p < 0.001). Compared with NCs, patients with CSU had higher levels of log-transformed total IgE (p < 0.001) and triglycerides (150.4 ± 98.7 vs. 104.2 ± 60.0 mg/dL, p < 0.001) and lower levels of HDL (55.7 ± 14.9 vs. 70.2 ± 27.0 mg/dL, p < 0.001), whereas no significant differences in total cholesterol and LDL were observed.

**Table 1:** Baseline characteristics of the study subjects

Characteristics	CSU (n = 283)	NC (n = 111)	*p* value
Age (year)	42.4 ± 11.7	43.9 ± 11.3	0.123
Female (%)	184 (65.0)	73 (65.8)	0.888
BMI (kg/m^2^)	23.5 ± 3.9	23.1 ± 2.8	0.864
Urticaria duration (month)	26.1 ± 51.8	NA	
UAS7 (0–42)	24.4 ± 10.3	NA	
Angioedema (%)	122 (45.7)	NA	
Aspirin intolerance (%)	63/148 (25.5)	NA	
ASST positivity (%)	81/268 (30.2)	NA	
ANA positivity (%)	58/271 (21.4)	NA	
Atopy (%)	150/277 (54.2)	NA	
Log[Total IgE (kU/L)]	2.09 ± 0.5	1.71 ± 0.6	< 0.001
ECP (kU/L)	21.7 ± 18.9	12.7 ± 9.6	< 0.001
Total cholesterol (mg/dL)	194.9 ± 36.8	188.4 ± 40.3	0.362
Triglycerides (mg/dL)	150.4 ± 98.7	104.2 ± 60.0	< 0.001
LDL (mg/dL)	111.9 ± 35.0	115.9 ± 35.0	0.319
HDL (mg/dL)	55.7 ± 14.9	70.2 ± 27.0	< 0.001
Complement 3 (mg/dL)	110.1 ± 20.8		
Complement 4 (mg/dL)	26.6 ± 7.9		

*p* values were obtained by Chi square test for categorical variables and Mann–Whitney U-test for continuous variables

*CSU* chronic spontaneous urticaria, *NC* healthy normal control, *BMI* body mass index, *NA* not assessable, *UAS7* urticaria activity score over 7 days, *ASST* autologous serum skin test, *ANA* anti-nuclear antibody, *ECP* eosinophilic cationic protein, *LDL* low density lipoprotein, *HDL* high density lipoprotein

H1RA non-responders were defined as patients whose symptoms could not be controlled by increasing doses of antihistamines (up to fourfold) within 3 months, and H1RA responders were defined as those who responded well to antihistamine treatment [11]. The clinical characteristics of patients according to H1RA response are summarized in Table 2. There were no differences in age (42.3 ± 12.0 vs. 42.6 ± 11.4 years, p = 0.665) or sex (female 65.7 vs. 64.6%, p = 0.852) between H1RA responders and non-responders, nor were there any significant differences in BMI (23.4 ± 4.1 vs. 23.6 ± 3.6, p = 0.577). The mean urticaria duration of the H1RA responder group was 28.8 ± 56.8 months, and that for the H1RA non-responder group was 22.1 ± 43.0 months (p = 0.289). No differences were observed in serum total IgE and complement levels or lipid profiles between the two groups.

**Table 2:** Clinical characteristics of H1RA responders and H1RA non-responders among CSU patients

Characteristics	H1RA responders (n = 169)	H1RA non-responders (n = 113)	*p* value
Age (years)	42.3 ± 12.0	42.6 ± 11.4	0.665
Female n (%)	111 (65.7)	73 (64.6)	0.852
BMI (Kg/m^2^)	23.4 ± 4.1	23.6 ± 3.6	0.577
Urticaria duration (months)	28.8 ± 56.8	22.1 ± 43.0	0.289
UAS7 (0–42)	24.4 ± 10.3	28.8 ± 9.3	< 0.001
Log[Total IgE (kU/L)]	2.07 ± 0.5	2.13 ± 0.5	0.414
ECP (kU/L)	20.8 ± 16.5	23.1 ± 22.4	0.956
Total cholesterol (mg/dL)	194.8 ± 37.1	195.5 ± 36.3	0.985
Triglycerides (ng/dL)	148.7 ± 108.3	153.5 ± 84.8	0.288
LDL (mg/dL)	113.0 ± 37.8	110.7 ± 31.2	0.909
HDL (mg/dL)	56.1 ± 14.8	55.5 ± 15.3	0.862
Platelet (*1000/μL)	262.8 ± 56.7	263.0 ± 56.3	0.654
Mean platelet volume	8.0 ± 0.8	8.0 ± 0.7	0.544
Complement 3 (mg/dL)	108.6 ± 17.6	112.2 ± 24.7	0.525
Complement 4 (mg/dL)	26.3 ± 7.4	27.0 ± 8.6	0.481
H1RA dose^a^ (mg/day)	21.0 ± 9.4	30.8 ± 13.3	< 0.001
Steroid use (%)	88 (52.1)	106 (93.8)	< 0.001
Cyclosporine use (%)	0	35 (31.0)	< 0.001
Omalizumab use (%)	0	38 (33.6)	< 0.001
PAF (pg/ml)	3804.6 [17.0–11716.4]	5426.4 [53.2–14768.3]	< 0.001
PAF-AH (ng/ml)	104.2 [2.6–296.5]	112.1 [4.4–256.9]	0.051

*p* values were obtained by Pearson’s Chi square test for categorical variables and Mann–Whitney U-test for continuous variables

Medication score, daily mean loratadine dose to control symptoms

*H1RA* histamine receptor 1 antagonist, *CSU* chronic spontaneous urticaria, *BMI* body mass index, *UAS7* sum of daily urticaria activity scores during the last week, *ASST* autologous serum skin test, *ANA* anti-nuclear antibody, *ECP* eosinophilic cationic protein, *LDL* low density lipoprotein, *HDL* high density lipoprotein, *PAF* platelet activating factor, *PAF*-*AH* platelet activating factor acetylhydrolase

^a^H1RA dose is presented as the mean daily H1RA prescription calculated as the loratadine equivalent dose for 3 months

H1RA non-responders had higher UAS7 scores than those who responded well to H1RA treatment (28.8 ± 9.3 vs. 24.4 ± 10.3, p < 0.001) at initial visits. They also had higher H1RA requirements calculated as the equivalent dose of loratadine (mg/day) (21.0 ± 9.4 vs. 30.8 ± 13.3, p < 0.001) for a subsequent 3 months after enrollment into the present study.

### Serum PAF and PAF-AH levels in CSU patients and NCs

**Fig. 1:**
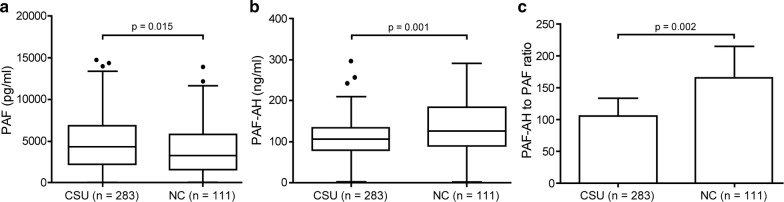
Serum levels of PAF (**a**) and PAF-AH (**b**) and PAF-AH to PAF ratio (**c**) in patients with CSU and NCs. *PAF* platelet activating factor, *PAF*-*AH* platelet activating factor acetylhydrolase, *CSU* chronic spontaneous urticaria, *NC* healthy normal control. *p* values were calculated by Mann–Whitney U-test

The median level of serum PAF was remarkably higher in CSU patients than in NCs (4368.9 [17.0–14768.3] vs. 3256.4 [27.1–13886.7] pg/ml, p = 0.015, Fig. 1a), while the median PAF-AH level was comparably lower in CSU patients than in NCs (105.6 [2.6–296.4] vs. 125.7 [2.0–291.1] ng/ml, p < 0.001, Fig. 1b). When we analyzed the ratio of PAF-AH to PAF, CSU patients had significantly lower PAF-AH/PAF ratios than NCs (Fig. 1c). However, there was no correlation between serum PAF and PAF-AH levels (r = 0.081, p = 0.175) in either CSU patients or NCs.

### Associations for serum PAF and PAF-AH levels with treatment response

**Fig. 2:**
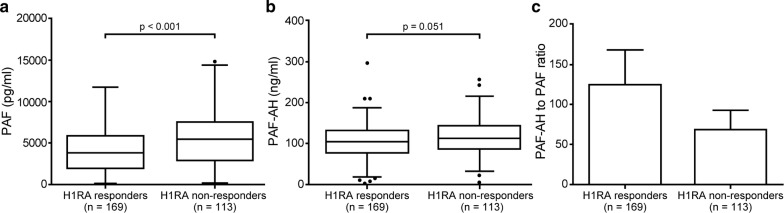
Serum levels of PAF (**a**) and PAF-AH (**b**) and PAF-AH to PAF ratio (**c**) in H1RA responders and H1RA non-responders among CSU patients. *PAF* platelet activating factor, *PAF*-*AH* platelet activating factor acetylhydrolase, *H1RA* histamine receptor 1 antagonist, *CSU* chronic spontaneous urticaria. *p* values were estimated using Mann–Whitney U-test

Interestingly, the median level of serum PAF was higher in H1RA non-responders than in H1RA responders (5426.3 [53.1–14768.3] vs. 3804.5 [17.0–11716.3] pg/mL, p < 0.001, Fig. 2a), while PAF-AH levels were not (104.2 [2.6–296.4] vs. 112.0 [4.4–256.8] ng/mL, p = 0.051, Fig. 2b). PAF-AH/PAF ratio was also higher in H1RA responders than in H1RA non-responders (124.5 ± 569.0 vs. 70.8 ± 278.8 ng/mL, p = 0.007, Fig. 2c).

**Fig. 3:**
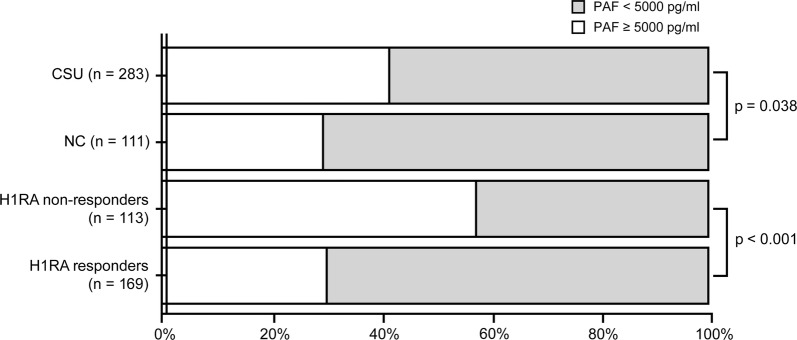
Prevalence of subjects with high PAF (≥ 5000 pg/ml) and low PAF (< 5000 pg/mL) in CSU patients versus NCs and H1RA responder versus non-responders. *PAF* platelet activating factor, *CSU* chronic spontaneous urticaria, *NC* healthy normal control, *H1RA* histamine receptor 1 antagonist

With a cut-off level for PAF of 5000 pg/mL obtained by ROC curve analysis, we divided CSU subjects and NCs with PAF levels ≥ 5000 pg/mL, particularly between H1RA responder and non-responders. Among CSU patients, 117 (41.3%) had PAF levels at least 5000 pg/mL in their sera, whereas the rate of high PAF (≥ 5000 pg/mL) in NCs was 29.7% (p = 0.038, Fig. 3). The rate of patients with high PAF was more frequent among H1RA non-responders than among H1RA responders (57.5 vs. 30.2%, p < 0.001, Fig. 3).

To identify predictors of responsiveness to antihistamine treatment in patients with CSU, we performed multivariate analysis using a generalized linear model containing age, sex, urticaria duration, UAS7, total IgE levels, BMI, ASST response, PAF-AH levels, and PAF ≥ 5000 pg/mL as covariates. Higher UAS7 scores (odds ratio 1.008, 95% confidence interval 1.001–1.016, p = 0.020) and PAF ≥ 5000 pg/mL (1.414, 1.217–1.642, p < 0.001) were found to be significant factors for discriminating H1RA non-responders among CSU patients (Table 3).

**Table 3:** Generalized linear model for predicting refractoriness to H1RA

Characteristics	Odds ratio (95% Cl)	*p* value
Age	1.004 (0.998–1.011)	0.209
Female	0.975 (0.827–1.148)	0.758
BMI	0.996 (0.973–1.019)	0.740
Urticaria duration	1.000 (0.998–1.001)	0.494
Total IgE	1.000 (1.000–1.000)	0.078
UAS7	1.008 (1.001–1.016)	0.020
ASST positivity	0.971 (0.825–1.142)	0.720
PAF (≥ 5000 pg/ml)	1.414 (1.217–1.642)	< 0.001
PAF-AH	1.000 (0.999–1.002)	0.582

*H1RA* histamine receptor 1 antagonist, *CI* confidence interval, *BMI* body mass index, *UAS7* urticaria activity score over 7 days, *ASST* autologous serum skin test, *PAF* platelet activating factor, *PAF*-*AH* platelet activating factor acetylhydrolase

With regard to serum PAF in CSU patients, no correlations with clinical parameters were observed (r = 0.042, p = 0.523 for BMI; r = 0.067, p = 0.264 for urticaria duration; r = 0.103, p = 0.084 for UAS7). Serum PAF-AH levels, however, were correlated positively with BMI (r = 0.131, p = 0.04) and negatively with urticaria duration (r = − 0.121, p = 0.045), while no correlation was found with UAS7 (r = 0.07, p = 0.215). PAF-AH showed positive correlations with C3 (r = 0.163, p = 0.007) and C4 (r = 0.185, p = 0.002) levels and total cholesterol (r = 0.231, p < 0.001) and triglyceride (r = 0.192, p = 0.014) levels in the sera of patients with CSU, whereas PAF did not (r = − 0.067, p = 0.275 for C3; r = − 0.031, p = 0.613 for C4; r = 0.029, p = 0.264 for total cholesterol; r = 0.133, p = 0.089 for triglyceride). No correlations were found between total IgE and ECP levels with either PAF (r = 0.032, p = 0.600 for total IgE; r = 0.123, p = 0.092 for ECP) or PAF-AH (r = − 0.023, p = 0.699 for total IgE; r = − 0.05, p = 0.451 for ECP) levels in the sera of CSU patients. Mean medication score (daily H1RA requirement defined as mean loratadine dose) was positively correlated with serum PAF-AH levels (r = 0.153, p = 0.019), but not with PAF levels (r = − 0.014, p = 0.835).

## Discussion

The present study demonstrates that PAF levels are increased and that PAF-AH levels are decreased in the sera from CSU patients, compared with those in healthy controls. These results are consistent with previous reports on increased PAF levels in anaphylactic patients [8, 12] and in asthmatics [13].

PAF is a rapid synthesizing, potent signaling phospholipid produced in response to stress, chemotactic factors, phagocyte stimulation, exogenous antigens or IgE/IgG, thrombin, IL-1, bacteria, or calcium ionophore [1]. PAF is synthesized and released from various immune cells, including neutrophils, monocytes, macrophages, platelets, eosinophils, and vascular endothelial cells [1], and accumulating evidence indicates that PAF is involved in inflammation and coagulation through its specific receptor expressed on a wide variety of cell surfaces [14]. PAF induces aggregation and activation of neutrophils to produce reactive oxygen species and eicosanoids, and PAF can augment IL-1, IL-6, and tumor necrosis factor (TNF) production via human cells. Meanwhile, IL-1 and TNF promotes the release of PAF in neutrophils, macrophages, and endothelial cells. Prior reports of perivascular cellular infiltration of neutrophils, eosinophils, T cells, and monocytes, as well as increased expression of IL-6 and TNF-α, in urticarial lesions [15] suggest that PAF could be a key player in the pathogenesis of CU. Indeed, intradermal injection of PAF has been found to induce transient wheals with increases in vascular permeability followed by neutrophil infiltration [16].

Previously, Amin et al. [17] reported that neutrophilic infiltrates on skin biopsy from CU patients is a marker of poor control. In the present study, a high level of serum PAF (> 5000 pg/mL) in CSU patients was identified as a novel predictor of a poor response to antihistamine treatment: higher PAF levels can lead to massive neutrophil chemotaxis and finally result in H1RA resistance. PAF has a short half-life (3–13 min) due to its fast metabolism by circulating PAF-AH, which also degrades oxidized phospholipids [8]. We found increased PAF levels and a lower PAF-AH to PAF ratio in CSU patients, compared with NCs. This suggests that impaired degradation of PAF caused by decreased PAF-AH levels might be a reason for increased PAF levels in CSU patients. However, we only found a marginal and statistically insignificant difference in PAF-AH levels between CSU patients and NCs and no difference according to H1RA treatment responses among CSU patients. The secretion of PAF-AH is regulated by various cytokines, as well as steroid hormones and gender. Pro-inflammatory cytokines known to be elevated in CSU patients, including IL-6, TNF-α, and interferon-gamma (IFN-γ), can stimulate macrophages to express both PAF-AH and complement as a physiologic response [18]. Accordingly, we noted significant correlations between PAF-AH and complements 3 and 4 in CSU patients in the present study. Moreover, we previously reported the co-existence of metabolic syndrome in severe refractory urticaria patients who had higher complement levels [19]. Additionally, increased levels of PAF-AH and LDL were found to be associated with abdominal adiposity in obese children and adults with metabolic syndrome [20, 21]. Interestingly, in the present study, significant correlations for BMI, total cholesterol, triglyceride, and complement levels with serum PAF-AH levels in patients with CSU were also observed. Consistent with previous reports in which estrogen was found to decrease PAF-AH levels, we also found that females had significantly lower levels of PAF-AH than males among both CSU patients and NCs [22]. However, because no difference was observed in PAF-AH levels between H1RA responders and non-responders, the pathologic effects of PAF-AH in CSU remains to be elucidated.

Research has demonstrated that recombinant PAF-AH attenuates inflammation in a variety of experimental models and that genetic deficiency of PAF-AH increases the severity of atherosclerosis and other inflammatory syndromes [18]. Correlating with severity, PAF-AH has been found to be decreased in both asthmatic patients and mice [22, 23]. Moreover, reports have indicated that human PAF is associated with increased nasal airway resistance to histamine and nasal symptoms [24, 25]. A PAFR deficient asthma mouse model showed reduced airway hyper-responsiveness [22, 26, 27], and studies have revealed correlations between increased PAF levels and anaphylaxis severity [8, 12]. Accordingly, inactivation of PAF or PAF receptor antagonist has been proven to prevent anaphylaxis in vivo [28, 29].

The estimated prevalence of CSU worldwide is around 0.5–1%, and 50% of CSU individuals do not achieve control with H1RA treatment [30]. While biomarkers to predict H1RA responsiveness in CSU patients would be helpful, evidence thereof is sparse. Meanwhile, an increase in serum levels of lipocalin-2 has been suggested as a useful biomarker with which to determine clinical responses to antihistamine treatment in CSU patients: this biomarker has also been shown to be correlated with metabolic syndrome and obesity [11, 31]. Additionally, D-dimer, fibrinogen, C-reactive protein, and erythrocyte sedimentation rate have been highlighted as promising biomarkers for predicting poor responses to antihistamine treatment [32]. Also, increases in IL-6 have been reported in refractory CU [33]. Notably, these features of activating coagulation and chronic low-grade inflammation, which are observed in severe CU, can be linked with increases in PAF levels and PAF-induced coagulation and inflammation.

Rupatadine is a long-acting H1-antihistamine that antagonizes PAF and has recently been shown to be effective in allergic diseases, including urticaria [34, 35]. It has been found to decrease the severity of peanut-induced anaphylaxis and to alleviate symptoms of allergic rhinoconjunctivitis and cold urticaria [36–38]. As indicated in multivariate analysis in the present study, higher UAS7 score and high PAF levels (PAF ≥ 5000 pg/mL) at baseline could be significant predictors of a poor response to H1RA treatment. Therapeutic strategies to inhibit PAF or to stimulate PAF-AH might be beneficial for H1RA refractory CSU patients.

## Conclusion

In conclusion, we found serum levels of PAF to be increased and PAF-AH levels to be decreased in CSU patients, compared with healthy controls. Accordingly, we propose that high levels of PAF in serum could be a potential predictor of refractoriness to antihistamine treatment in CSU patients. For severe patients with high UAS7 scores, increasing antihistamines with the addition of a PAF antagonist may help to achieve better control of CU earlier. Further studies are required to elucidate PAF and PAF-AH signaling and therapeutic modulation thereof in CSU.

## Data Availability

Not applicable.

## Abbreviations

ANA: anti-nuclear antibody
ASST: autologous serum skin test
BMI: body mass index
CI: confidence interval
CSU: chronic spontaneous urticaria
CU: chronic urticaria
ECP: eosinophil cationic protein
ELISA: enzyme-linked immunosorbent assay
H1RA: histamine receptor 1 antagonist
HDL: high density lipoprotein
IQR: interquartile range
LDL: low density lipoprotein
NA: not assessible
NC: healthy normal control
PAF: platelet activating factor
PAF-AH: platelet activating factor acetylhydrolase
ROC: receiver operating characteristic
TNF: tumor necrosis factor
UAS: urticaria activity score
UAS7: urticaria activity score over 7 days
